# Impact of partial substitution of cisplatin with cyclophosphamide on acute toxicities in standard-risk medulloblastoma

**DOI:** 10.1007/s11060-025-05098-7

**Published:** 2025-06-10

**Authors:** Sarah Magdy Metwally, Moatasem El-Ayadi, Eslam Maher, Mohamed Sherif El-Minawi, Mohamed S. Zaghloul, Hala Taha, Sherif Aboulnaga, Iman Sidhom

**Affiliations:** 1 Department of Pediatric Oncology, National Cancer Institute, Cairo University, Cairo, Egypt; 2 Department of Pediatric Oncology, Children’s Cancer Hospital Egypt (CCHE-57357), Cairo, Egypt; 3 Department of Clinical Research, Children’s Cancer Hospital Egypt (CCHE-57357), Cairo, Egypt; 4 Department of Surgery and Cancer, Faculty of Medicine, Imperial College London, London, UK; 5 Department of Otolaryngology, Kasr El-Ainy School of Medicine, Cairo University, Cairo, Egypt; 6 Children’s Cancer Hospital Egypt (CCHE-57357), Cairo, Egypt; 7 Department of Radiation Oncology, Children’s Cancer Hospital Egypt (CCHE-57357), Cairo, Egypt; 8 Department of Radiation Oncology, National Cancer Institute, Cairo University, Cairo, Egypt; 9 Department of Pathology, Children’s Cancer Hospital Egypt (CCHE-57357), Cairo, Egypt; 10 Department of Pathology, National Cancer Institute, Cairo University, Cairo, Egypt

**Keywords:** Medulloblastoma, Chemotherapy, Cisplatin, Cyclophosphamide, Toxicity, Ototoxicity

## Abstract

**Purpose:**

Medulloblastoma (MB) treatment includes surgery, irradiation, and chemotherapy (CT). Cisplatin-based regimens for standard-risk (SR) MB are effective but associated with significant toxicities, particularly ototoxicity. This study compares the toxicity profiles of two CT regimens, focusing on grade ≥ 3 ototoxicity, hematologic, hepatic, renal, and neurologic toxicities.

**Methods:**

This study included SR-MB patients aged 3–18 years. Cohort A (2016–2019) received adjuvant CT adopted from the Children’s Oncology Group (COG) A9961 Regimen A protocol, with data collected retrospectively. Cohort B (2020–July 2022) received CT adopted from the ACNS0331 protocol, with data collected prospectively. Toxicities were assessed and graded using the Common Terminology Criteria for Adverse Events (CTCAE) v5.0.

**Results:**

A total of 168 patients aged 3 to 18 years were enrolled, 112 (67%) in cohort A and 56 (33%) in cohort B. Grade ≥ 3 ototoxicity was significantly higher in cohort A (24% vs. 3.6%, *p* < 0.001). Neurotoxicity occurred in 26% vs. 12.5% (*p* = 0.046). Anemia and thrombocytopenia in 71% vs. 52% (*p* = 0.04). Febrile neutropenia was more common in cohort B (66% vs. 38%, *p* < 0.001). No significant differences were found in grade ≥ 3 leukopenia, nephrotoxicity or hepatotoxicity. The 2-year overall survival was 96.4% (95% CI: 93.1–99.9) in cohort A vs. 86.6% (95% CI: 77.8–96.4) in cohort B (*p* = 0.11). Event-free survival was 92.9% (95% CI: 88.2–97.8) vs. 86.8% (95% CI: 78-96.4) (*p* = 0.29).

**Conclusion:**

Partial substitution of cisplatin with cyclophosphamide showed a better toxicity profile, particularly for ototoxicity and neurotoxicity, with no significant difference in survival.

**Supplementary Information:**

The online version contains supplementary material available at 10.1007/s11060-025-05098-7.

## Introduction

Medulloblastoma (MB) is the most common malignant brain tumor in pediatric patients, accounting for 25% of childhood brain tumors and over 40% of posterior fossa tumors [1–3]. Treatment for standard-risk (SR) MB includes maximal surgical resection followed by craniospinal radiotherapy (RT) with a boost to the posterior fossa or involved field [4–7]. A variety of different chemotherapeutic agents have been utilized and encouraging results have been obtained with the use of vincristine (VCR) during RT, followed by cycles of lomustine (CCNU), VCR, and cisplatin which results in a 5-year survival rate of up to 85% for SR-MB patients [8–11]. While this combined-modality treatment regimen has substantially improved the cure rate, survivors suffer from long-term toxic side effects that often seriously affect their quality of life (QoL) [12–15].

The Children’s Oncology Group (COG) A9961 trial showed comparable survival outcomes between the CCNU and cyclophosphamide arms, both associated with tolerable but notable toxicity, including severe (grade 3 or 4) ototoxicity in a quarter of patients due to cisplatin administered at a cumulative dose of 600 mg/m² [8]. Other studies, such as HIT’91, revealed that chemotherapy (CT) dose modifications are often required due to toxicities, particularly in older children [16].

The subsequent COG-ACNS0331 trial used a different CT regimen with 9 cycles (AABAABAAB), including VCR, cisplatin, and CCNU (cycle A), and VCR with cyclophosphamide (cycle B), with a reduced cumulative cisplatin dose of 450 mg/m². Despite the dose reduction, survival outcomes were similar to earlier trials [4], and the lower cisplatin dose is expected to reduce the incidence of severe ototoxicity.

In this study, we compared the acute toxicity profiles of CT regimens from both the COG-A9961 and COG-ACNS0331 trials by analyzing the frequency of grade ≥ 3 toxicities, including ototoxicity, hematologic toxicity, hepatotoxicity, nephrotoxicity, and neurotoxicity, as well as the impact on hospital stay duration and the need for dose reduction or cycle omission.

## Methods

### Study design and population

This ambidirectional cohort study included patients aged 3 to 18 years diagnosed with histologically confirmed MB, meeting the standard-risk criteria; no evidence of tumor dissemination, less than 1.5 cm² of residual tumor after surgery, and non-anaplastic histology [4]. Patients enrolled at Children’s Cancer Hospital Egypt (CCHE) from January 2016 to July 2022, with no prior exposure to RT or CT, normal baseline hearing in at least one ear, and normal kidney function, were included. Between 2016 and 2019, patients (cohort A) received their treatment according to the COG-A9961 CT, regimen A protocol, with cumulative doses of 48 mg/m² of VCR, 600 mg/m² of cisplatin, and 600 mg/m² of CCNU [8] (Supplementary Fig. 1). In January 2020, our institutional protocol was shifted to the COG-ACNS0331 regimen (cohort B), with cumulative doses of 45 mg/m² of vincristine, 450 mg/m² of cisplatin, 450 mg/m² of CCNU, and 6000 mg/m² of cyclophosphamide [4] (Supplementary Fig. 2). Data on presenting symptoms, demographics, and toxicity were collected retrospectively for cohort A and prospectively for cohort B. All patients were followed longitudinally for at least 18 months to monitor for recurrence or death.

### Data collection

The electronic medical files were reviewed for patients’ demographic data (age and gender), presenting symptoms, magnetic resonance imaging (MRI) scans of brain and spine obtained pre- and immediate post-operatively, after irradiation, during CT, and post-treatment, initial cerebrospinal fluid (CSF) cytology, surgical excision, histological subtypes, RT dose and field, CT regimen received (including dose modifications and cycle omissions), and days of hospitalization. Baseline/follow-up audiometry and creatinine clearance were collected before each cycle of cisplatin-containing CT and at the end of treatment. Pure-tone audiometry (PTA) was used as the standard method for hearing evaluation at our institution. Most patients underwent PTA; however, for younger children (particularly those aged 3–5 years) and non-cooperative patients, auditory brainstem response (ABR) testing was used as an objective alternative. Other laboratory tests, including complete blood counts, liver and kidney function tests, and electrolyte levels (magnesium, phosphorus, potassium, and sodium), were measured before and weekly for the first three weeks post each cycle. Finally, any clinical data documenting treatment-related neurotoxicity, such as constipation, peripheral neuropathy, and ptosis, were also recorded. Diagnosis of neurotoxicity was primarily clinical; however, for patients presenting with limb weakness, nerve conduction velocity (NCV) studies were selectively performed.

The instructions for drug administration, scheduling, and toxicity modifications were applied according to our institutional guidelines, which are adopted from COG protocols [4, 8].

### Radiotherapeutic procedures

Both groups were referred for irradiation 4–6 weeks post-surgery. All children underwent computed tomography and MRI simulation. The clinical target volume included the posterior fossa (2016–2018) or the tumor bed with a 1 cm safety margin (2019–2022). Craniospinal irradiation was delivered at a dose of 2340 cGy, with a boost of 3240 cGy to the posterior fossa or tumor bed, for a total dose of 5580 cGy. Treatment was administered using Volumetric Modulated Arc Therapy (VMAT) with daily cone-beam computed tomography verification.

### Toxicity analysis

The occurrence, timing, type, and grade of different toxicities, including neurotoxicity, ototoxicity, nephrotoxicity, hepatotoxicity, and hematological toxicity, were recorded. Common Terminology Criteria for Adverse Events (CTCAE) version 5.0 was used to assess and grade adverse events [17]. Regarding ototoxicity, the two groups were compared at the same specific time points: baseline (post-induction RT and before adjuvant CT(, cycles 1–3, cycles 4–6, and cycles 7–9 (if applicable).

### Statistical analysis

Cumulative toxicity rates were measured as relative frequencies per patient and per cycle. The denominator for the number of episodes per cycle was calculated as the actual number of cycles, accounting for omitted cycles. Frequencies were compared between the two protocols using Pearson’s Chi-square test and Fisher’s exact test. Numerical data were compared using the t-test or the Wilcoxon rank sum test”. Survival analysis was performed using the Kaplan–Meier method. The 95% confidence intervals were calculated based on the cumulative hazard function, derived from the log of the survival estimates. Overall survival (OS) was defined as time from registration to death or last follow-up, while event-free survival (EFS) was defined as the time until recurrence, death, or last follow-up. The log-rank test was used to compare survival probabilities. All tests are two-sided, with alpha set at 0.05. The analysis was done using R Project for Statistical Computing (RRID: SCR_001905), version 4.1.0.

### Ethical committee approval

The study was approved by the Institutional Review Board (IRB) at CCHE (IRB number: 52/2020). No additional consent was required, as patients had already signed institutional chemotherapy-specific consent. Privacy and confidentiality of patients’ data were ensured.

## Results

### Patient characteristics

A total of 168 SR-MB patients were included in the study, 112 patients (66.7%) in cohort A and 56 (33.3%) in cohort B. The median age at diagnosis for all cases was 7.94 years (IQR: 5.70-10.59), with male: female ratio of 1.9:1. Headache and vomiting were the most common presentation in 98% of patients, followed by ataxia in 57%, squint in 16%, and other symptoms in 7.1%. A considerable proportion of patients presented late with advanced signs of increased intracranial tension, necessitating urgent ventriculoperitoneal (VP) shunt insertion in 95% of patients (*n* = 160).

The most common histological subtype was the classic variant (*n* = 133; 79%), followed by desmoplastic/nodular variant (*n* = 30; 18%). Age, gender, and histological subtypes were similarly distributed between the two cohorts.

Molecular subtyping by immunohistochemistry was available for 88 cases (52.3%): 30.3% of cohort A and 96.4% of cohort B. These tumors were classified as wingless (WNT) activated, sonic hedgehog (SHH) activated, or non-WNT non-SHH. Nearly half of the analyzed cases (47.7%) were of the non-WNT non-SHH subtype, however, treatment was not based on molecular subtypes. Baseline characteristics for both cohorts are summarized in Table 1.

**Table 1:** Baseline patients’ characteristics

Characteristic	Total, *N* = 168	A9961, *N* = 112 (cohort A)	ACNS0331, *N* = 56 (cohort B)	*P*
**Gender**				0.8
Female	58 (35%)	38 (34%)	20 (36%)	
Male	110 (65%)	74 (66%)	36 (64%)	
**Age**				0.3
Median (IQR)	7.94 (5.70-10.59)	8.15 (6.33–10.59)	7.85 (4.92–10.32)	
Range	3.1–17.9	3.1–16.7	3.1–17.9	
**Initial presentation**				
Headache & vomiting	165 (98%)	109 (97%)	56 (100%)	0.6
Ataxia	96 (57%)	56 (50%)	40 (71%)	0.008
Squint	27 (16%)	13 (12%)	14 (25%)	0.026
Others	12 (7.1%)	9 (8.0%)	3 (5.4%)	0.8
**Pathology**				0.5
Classic variant	133 (79%)	91 (81%)	42 (75%)	
Nodular/ desmoplastic	30 (18%)	17 (15%)	13 (23%)	
MB with melanotic differentiation	2 (1.2%)	2 (1.8%)	0 (0%)	
MB with extensive nodularity	1 (0.6%)	1 (0.9%)	0 (0%)	
MB, NOS	2 (1.2%)	1 (0.9%)	1 (1.8%)	
**Molecular subtyping** ^a^				0.057
WNT activated	12 (13.6%)	7 (21%)	5 (9.3%)	
SHH activated	34 (38.6%)	16 (47%)	18 (33.3%)	
Non-WNT, non-SHH	42 (47.7%)	11 (32%)	31 (57.4%)	

^a^ Evaluable patients for molecular subtyping were 88 patients: 34 patients in cohort A and 54 patients in cohort B

Abbreviations: SD, standard deviation; IQR, interquartile range; NOS, not otherwise specified; MB, medulloblastoma; WNT, wingless; SHH, sonic hedgehog

### Treatment- related toxicities

**Fig. 1:**
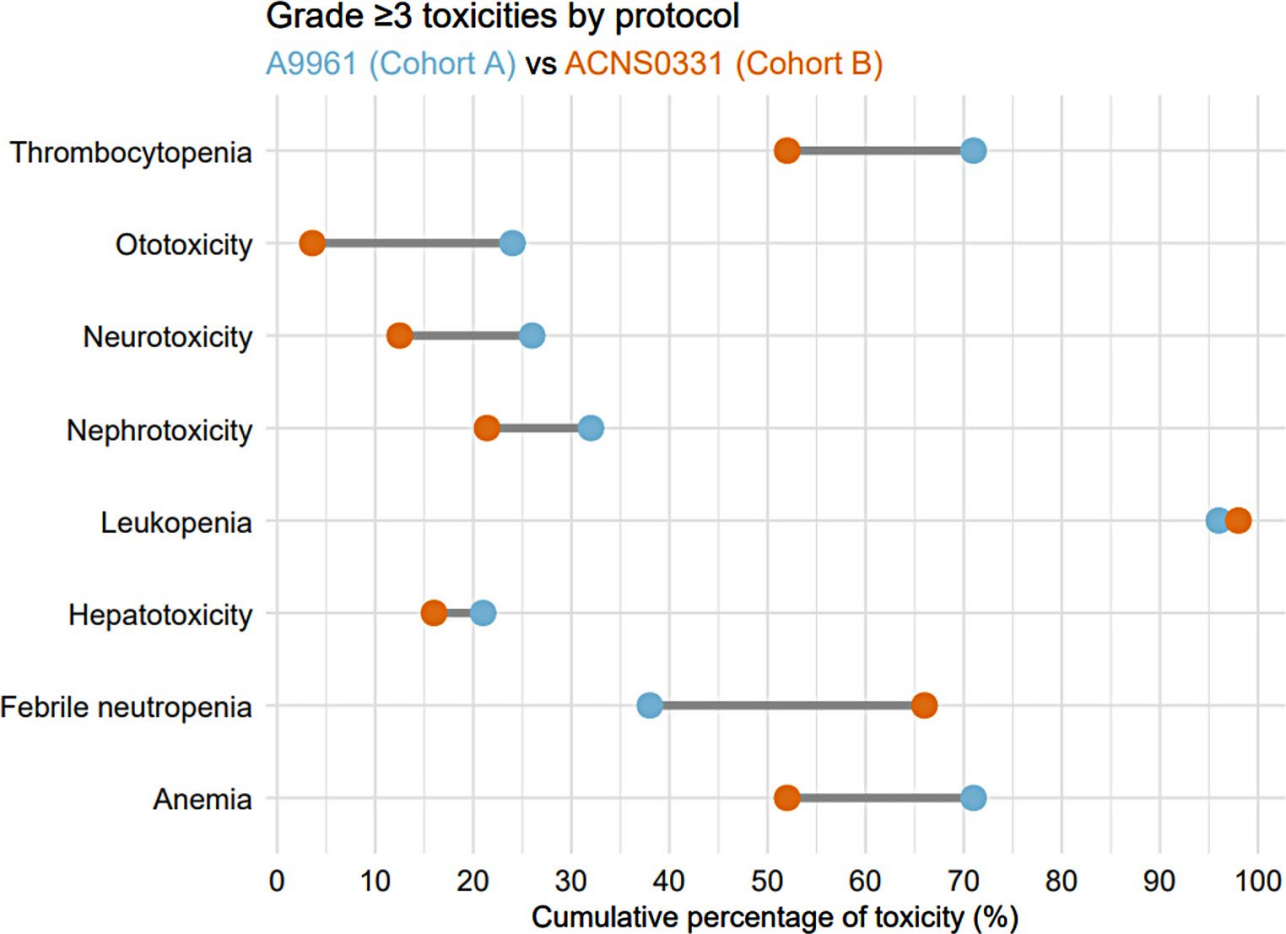
Comparison of grade ≥ 3 toxicities between both cohorts

Various types of toxicities encountered in both cohorts are illustrated in Fig. 1 and Supplementary Table 1. A comparative summary of the total number of toxicity episodes- specifically hematologic, nephrotoxic, and hepatotoxic events- documented across all evaluable treatment cycles is provided in Table 2.

**Table 2:** Comparative summary of hematologic, nephrotoxic, and hepatotoxic events across treatment cycles

Toxicity Type (grade ≥ 3)	Total Cycles, *n* = 1328 (%)	Cohort A *n* = 855^a^ (%)	Cohort B *n* = 473^a^ (%)	*P* value
Nephrotoxicity	67 (5%)	51 (6%)	16 (3%)	0.18
Hepatotoxicity	46 (3%)	36 (4%)	10 (2%)	0.05
Leukopenia	898 (68%)	580 (68%)	318 (67%)	0.8
Thrombocytopenia	327 (25%)	250 (29%)	77 (16%)	< 0.001
Anemia	423 (32%)	300 (35%)	123 (26%)	< 0.001
Fever & Neutropenia	137 (10%)	69 (8%),	68 (14%)	< 0.001

^a^ Cohort A included 896 treatment cycles, with 41 cycles unavailable (NA). Cohort B included 504 cycles, with 31 cycles NA. Unavailable cycles were due to various reasons, including treatment omission secondary to severe toxicity, treatment abandonment, protocol deviation due to disease progression/relapse, or patient death

### Neurotoxicity

Grade ≥ 3 vincristine-induced neurotoxicity (VIN) occurred in 36 patients (21%), of which 47% (*n* = 17) occurred during RT. The manifestations of VIN were mainly foot drop in 20 patients, ptosis in 14, and limb weakness in 5 patients. Neurotoxicity events in cohort A (*n* = 29, 25.8%), were grade 3 in 25 patients, grade 4 in 3 patients, and grade 5 in one patient. While in cohort B, neurotoxicity occurred in 7 patients (12.5%) and all of them were grade 3. VIN was statistically significantly higher in cohort A, *p* = 0.046. Almost all patients who experienced neurotoxicity needed CT dose modifications (*n* = 34), with 3 patients in cohort A needing the omission of one or two CT cycles.

### Ototoxicity

The mean cumulative cisplatin dose received was 511.36 mg/m^2^ (SD, 80.67) in cohort A and 382.70 mg/m^2^ (SD, 88.22) in cohort B. The median dose was 525 mg/m^2^ (IQR, 450-595.31) in cohort A (87.5% of total cumulative planned dose) and 412.50 mg/m^2^ (IQR, 351.56–450) in cohort B (91.7% of total cumulative planned dose), *p* < 0.001. Overall, 69% of patients experienced grade ≥ 1 ototoxicity, with a higher incidence in cohort A (74%) compared to cohort B (59%), *p* = 0.045. Severe (grade 3 or 4) ototoxicity occurred in 17.3% of patients, a frequency significantly higher in cohort A (24%) than in cohort B (3.6%), *p* < 0.001. Incidence of severe ototoxicity every 3 maintenance cycles is summarized in Supplementary Table 2.

Chemotherapy dose modifications due to ototoxicity were required in 74% of cohort A and one or more cycles were omitted due to grade 4 ototoxicity in four patients. In cohort B, 59% required dose adjustments, but none discontinued CT due to ototoxicity.

### Hematological toxicity

Grade ≥ 3 leukopenia was highly prevalent, affecting 96.4% of patients across both protocols. Among the 1328 evaluable cycles, 898 episodes were reported, with no significant difference in episode frequency between the two protocols, 580/855 episodes (68%) in cohort A, and 318/473 episodes (67%) in cohort B, *p* = 0.8.

Grade ≥ 3 thrombocytopenia was significantly more common in cohort A, occurring in 71% of patients compared to 52% in cohort B (*p* = 0.04). Similarly, higher thrombocytopenic episodes occurred in cohort A, 29% vs. 16%, respectively, *p* < 0.001.

Anemia grade ≥ 3 also occurred more frequently in cohort A, affecting 71% of patients compared to 52% in cohort B (*p* = 0.04). The number of anemia episodes was significantly higher in cohort A being reported in 35% of the cycles, compared to 26% in cohort B, *p* < 0.001.

Overall, grade ≥ 3 fever and neutropenia occurred in 47% of patients, but the incidence was much lower in cohort A than in cohort B (38% vs. 66%, respectively, *p* < 0.001).

The rate of fever and neutropenia episodes was higher in cohort B (14.4% vs. 8.1%, *p* < 0.001); however, this did not affect the length of hospital stay (LoS), which averaged 11 days for cohort A and 12 days for cohort B (*p* = 0.95). All fever and neutropenia episodes in cohort B were grade 3.

### Renal and urinary toxicity

- **Nephrotoxicity**

Forty-eight patients (28.6%) developed grade ≥ 3 nephrotoxicity in total, corresponding to 67 episodes throughout treatment. Most episodes were grade 3 (*n* = 58) rather than grade 4 (*n* = 9). Electrolyte imbalances were reported in 89.6%, and 10.4% involved elevated serum creatinine. Only one episode required CT modification, occurring in cohort A.

Patients in cohort A had a higher incidence of nephrotoxicity (32%) than cohort B (21%), but the difference was not statistically significant, *p* = 0.15. This corresponded to 6% of the cycles in cohort A and 3.4% in cohort B, *p* = 0.18.

- **Urinary bladder toxicity**

Three patients in cohort B (5%) experienced hemorrhagic cystitis after receiving cyclophosphamide containing CT cycles, all of them were grade 3.

### Hepatotoxicity

The overall rate of grade ≥ 3 hepatotoxicity in our study was 19%, with no significant difference between the two regimens: 21% in cohort A and 16% in cohort B (*p* = 0.3). However, cohort A had a higher proportion of hepatotoxicity episodes than cohort B (4.2% vs. 2.1% of cycles, respectively), with this difference approaching statistical significance (*p* = 0.05). All hepatotoxicity episodes were grade 3, and none required CT modifications.

The number of chemotherapy cycles omitted due to each toxicity is summarized in Table 3.

**Table 3:** Chemotherapy cycles omitted due to various toxicities

Causes of CT cycles omission	Cohort A		Cohort B	
	No. of patients (%)	No. of cycles	No. of patients (%)	No. of cycles
Neurotoxicity	3 (2.7%)	5	-	-
Ototoxicity	4 (3.5%)	7	-	-
Hematological toxicity	6 (5.3%)	9	2 (3.6%)	2
Nephrotoxicity	1 (0.9%)	3	-	-
Hepatotoxicity	-	-	-	-

### Length of hospital stay due to toxicity

There was no difference in the duration of hospital admissions due to toxicities per episode between the two cohorts. The mean duration was 12 (± 8) days for cohort A and 12 (± 9) days for cohort B, the median duration was 10 (IQR, 6–16) days and 10 (IQR, 5–14) days, respectively, *p* = 0.6.

### Survival analysis

**Fig. 2:**
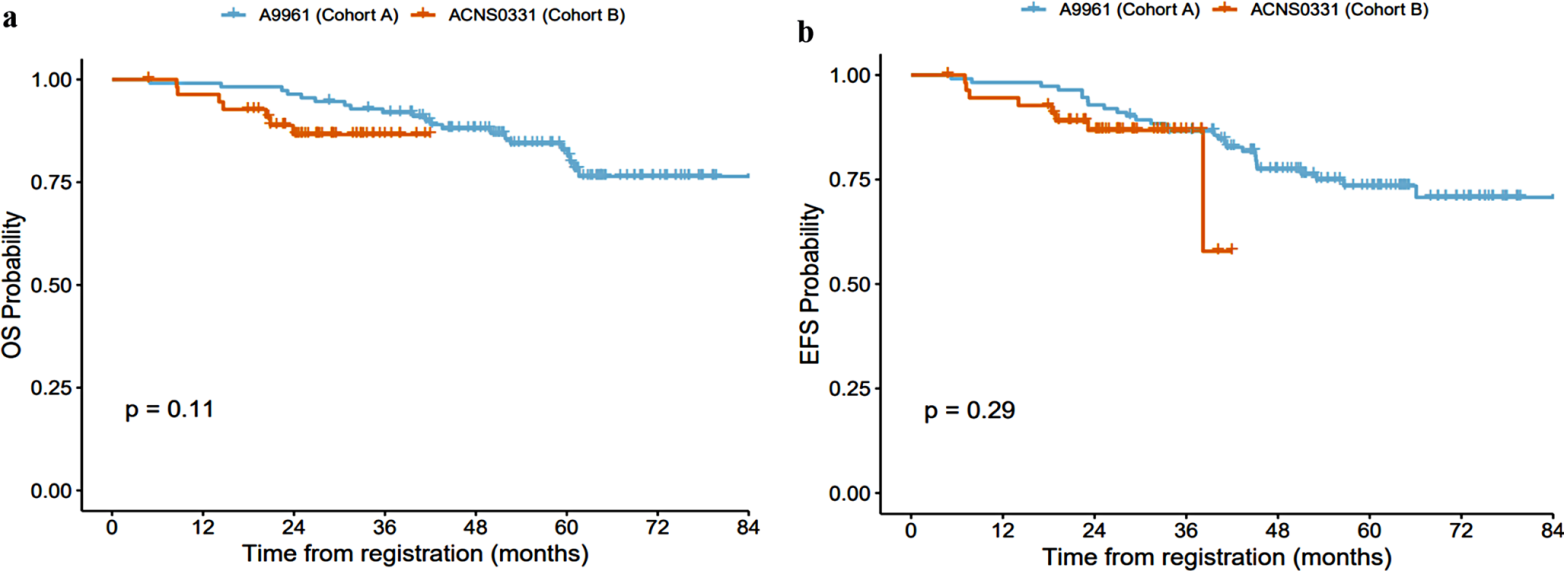
**a** Overall Survival for Both Regimens. **b** Event-Free Survival for Both Regimens

The median follow-up for cohort A was 62.8 months (range 5-85.6) and 27.7 months (range 4.8–42) for cohort B. The 2-year OS was 96.4% (95% CI: 93.1–99.9) for cohort A and 86.6% (95% CI: 77.8–96.4) for cohort B, *p* = 0.11 (Fig. 2a). Similarly, the 2-year EFS were 92.9% (95% CI: 88.2–97.8), and 86.8% (95% CI: 78-96.4), respectively, *p* = 0.29 (Fig. 2b). The differences in survival between the two groups were not statistically significant, however, it is noteworthy that cohort B had a smaller sample size and a shorter median follow-up period.

## Discussion

Cisplatin-based CT regimens for SR-MB have significantly improved survival rates, but toxicity remains a key concern, particularly ototoxicity, which can severely impact patients’ QoL [3, 18–21]. To evaluate the impact of reduced cumulative cisplatin dosing in adjuvant chemotherapy for SR-MB, we compared two regimens: cohort A received higher cumulative doses of cisplatin and CCNU, while cohort B received lower doses of both agents with the addition of cyclophosphamide. The present analysis focused on the cohorts’ differences in ototoxicity and other treatment-related adverse effects.

Treatment with VCR is limited by progressive VIN, a dose-limiting side effect that can compromise QoL and lead to discontinuation of VCR [22–25]. Neurotoxicity events were more frequent and severe in cohort A, likely due to higher VCR doses, particularly during RT. Comparing neurotoxicity across studies is challenging due to inconsistent assessment methods and the absence of an established gold standard, which hinders accurate comparisons regarding the incidence, prevention, and treatment of VIN [22, 26]. Some studies indicated that molecular markers, genetic polymorphisms in VCR metabolism pathways, may help predict which patients are at risk for severe neuropathy, potentially aiding individualized treatment [22, 27].

Ototoxicity is a common side effect of cisplatin, affecting up to 77% of pediatric cancer patients. This irreversible sensorineural hearing loss (SNHL) can develop during or after treatment, affecting one or both ears, and initially affecting higher frequencies [28–31].

In the COG-A9961 study, only 20% of patients received the maximum cisplatin dose (600 mg/m²) due to toxicity. The average dose administered was 487.5 mg/m², and this lower dose did not appear to affect survival outcomes. The study reported a high incidence of severe ototoxicity, prompting the recommendation of stricter cisplatin dose adjustments to minimize SNHL [8]. In present study, chemotherapy dose modifications due to ototoxicity were required in 74% of cohort A, and one or more cycles were omitted due to grade 4 ototoxicity in four patients. In contrast, only 59% of patients in cohort B required dose adjustments, and none discontinued CT due to ototoxicity.

The overall rate of ototoxicity was higher in cohort A than in cohort B. Notably, the incidence of severe ototoxicity was significantly greater in cohort A (24%) compared to cohort B (3.6%). Despite more frequent dose modifications in cohort A, severe ototoxicity remained higher, suggesting a potential benefit from the reduced cisplatin exposure in cohort B.

Other factors, such as differences in RT, may have contributed to the observed ototoxicity. All patients in our study received photon-based RT. In terms of radiation fields, patients in cohort A predominantly received posterior fossa RT, which may have resulted in greater cochlear exposure compared to the involved field RT consistently used in cohort B. Further research is needed to explore the association between cochlear radiation dose and ototoxicity.

Studies have shown that the use of amifostine or sodium thiosulfate can reduce cisplatin-induced SNHL without compromising tumor control [32–35]. These findings suggest the potential benefit of integrating oto-protective agents into MB treatment protocols. Notably, none of the patients in our cohorts received oto-protection due to its unavailability.

Cisplatin ototoxicity worsens over time due to cisplatin trapped in the inner ear, so long-term audiometric monitoring remains essential for all patients receiving cisplatin-based therapies [31, 36].

Regarding hematologic toxicity, grade 3 or 4 leukopenia was observed in nearly all patients in our study. Both anemia and thrombocytopenia were more prevalent in cohort A, while cohort B, who received cyclophosphamide, experienced higher rates of febrile neutropenia. Importantly, all cases of febrile neutropenia in cohort B were grade 3, without associated mortality or complications, indicating good overall tolerability of the regimen.

The introduction of outpatient cyclophosphamide cycles has reduced the duration of hospital admissions needed for CT in cohort B compared to cohort A. Although cohort B experienced a higher frequency of fever and neutropenia, our study revealed no significant difference in the total duration of hospital stay due to toxicity between the two cohorts. Thus, both cohorts had a similar LoS for toxicity, however, cohort B had a shorter overall LoS, which can translate into lower treatment cost and fewer delays due to waiting lists for admission.

This study had several limitations. The retrospective component may have introduced reporting bias, while the prospective arm was limited by a relatively small sample size and shorter follow-up duration. Molecular subtyping was lacking in a substantial portion of cohort A, as routine testing was not implemented at our institution until 2018. Additionally, the COVID-19 pandemic disrupted access to audiology services for cohort B, leading to a five-month gap in formal audiometric assessments. To mitigate this, monitoring intervals were extended to every three cycles to detect potential SNHL.

In terms of survival outcomes, both cohorts achieved over 85% overall and event-free survival rates at two years, with a trend toward better survival in cohort A that was not statistically significant. Longer follow-up periods and larger sample size are needed to confirm comparability. Additionally, we could not analyze the association between molecular subgroups and survival outcomes due to the unavailability of molecular data in two-thirds of cohort A.

Molecular subgroup analysis has demonstrated significant survival differences among MB subgroups, with the WNT group showing the most favorable outcomes, followed by group 4, SHH, and group 3 [4, 37–40]. These findings support the value of molecular stratification in guiding personalized therapy, possibly improving survival outcomes and reducing treatment-related toxicities. Nonetheless, in resource-limited settings where molecularly tailored treatments are unavailable, standard CT for SR-MB remains the recommended approach.

It remains challenging to recommend one CT regimen over another as the preferred standard. However, our results suggest that partial substitution of cisplatin with cyclophosphamide is both effective and well-tolerated.

Going forward, extended follow-up and larger-scale studies are essential to validate these findings, assess long-term toxicities- including the risk of secondary malignancies- and optimize treatment strategies. Ultimately, the goal remains to sustain high survival rates while minimizing long-term side effects and improving patients’ quality of life.

## Electronic supplementary material

Below is the link to the electronic supplementary material.

Supplementary Material 1

## Data Availability

The datasets generated and/or analyzed during the current study are available from the corresponding author on reasonable request.
