# Supplementary material for: Impact of partial substitution of cisplatin with cyclophosphamide on acute toxicities in standard-risk medulloblastoma
Source: J Neurooncol. 2025 Jun 10;174(3):689–97. doi: 10.1007/s11060-025-05098-7 (PMC12263714; doi:10.1007/s11060-025-05098-7)
Supplement: Supplementary file 1 — Supplementary Material 1 [file 11060_2025_5098_MOESM1_ESM.pdf]

Supplementary Information

Title:

Impact of Partial Substitution of Cisplatin with Cyclophosphamide on Acute Toxicities in Standard-Risk Medulloblastoma

Journal of Neuro-Oncology

Author information:

1. **Sarah Magdy Metwally (First author, Corresponding author)**
   Department of Pediatric Oncology, Children's Cancer Hospital Egypt (CCHE-57357), Cairo, Egypt
   Department of Pediatric Oncology, National Cancer Institute, Cairo University, Cairo, Egypt
2. **Moatasem El-Ayadi (Co–first author)**
   Department of Pediatric Oncology, Children's Cancer Hospital Egypt (CCHE-57357), Cairo, Egypt
   Department of Pediatric Oncology, National Cancer Institute, Cairo University, Cairo, Egypt
3. **Eslam Maher**
   Department of Clinical Research, Children's Cancer Hospital Egypt (CCHE-57357), Cairo, Egypt
   Department of Surgery and Cancer, Imperial College London, London, United Kingdom
4. **Mohamed Sherif El-Minawi (Deceased)**
   Department of Otolaryngology, Kasr El-Ainy School of Medicine, Cairo University, Cairo, Egypt
   Children's Cancer Hospital Egypt (CCHE-57357), Cairo, Egypt
5. **Mohamed S. Zaghloul**
   Department of Radiation Oncology, Children's Cancer Hospital Egypt (CCHE-57357), Cairo, Egypt
   Department of Radiation Oncology, National Cancer Institute, Cairo University, Cairo, Egypt
6. **Hala Taha**
   Department of Pathology, Children's Cancer Hospital Egypt (CCHE-57357), Cairo, Egypt
   Department of Pathology, National Cancer Institute, Cairo University, Cairo, Egypt
7. **Sherif Aboulnaga**
   Department of Pediatric Oncology, Children's Cancer Hospital Egypt (CCHE-57357), Cairo, Egypt
   Department of Pediatric Oncology, National Cancer Institute, Cairo University, Cairo, Egypt
8. **Iman Sidhom****
   Department of Pediatric Oncology, Children's Cancer Hospital Egypt (CCHE-57357), Cairo, Egypt
   Department of Pediatric Oncology, National Cancer Institute, Cairo University, Cairo, Egypt

Corresponding Authors:

Sarah Magdy Metwally

Email: sarah.magdy@57357.org; sarah.magdy888@gmail.com

Iman Sidhom

Email: iman.sidhom@57357.org; ImanSidhom@yahoo.com

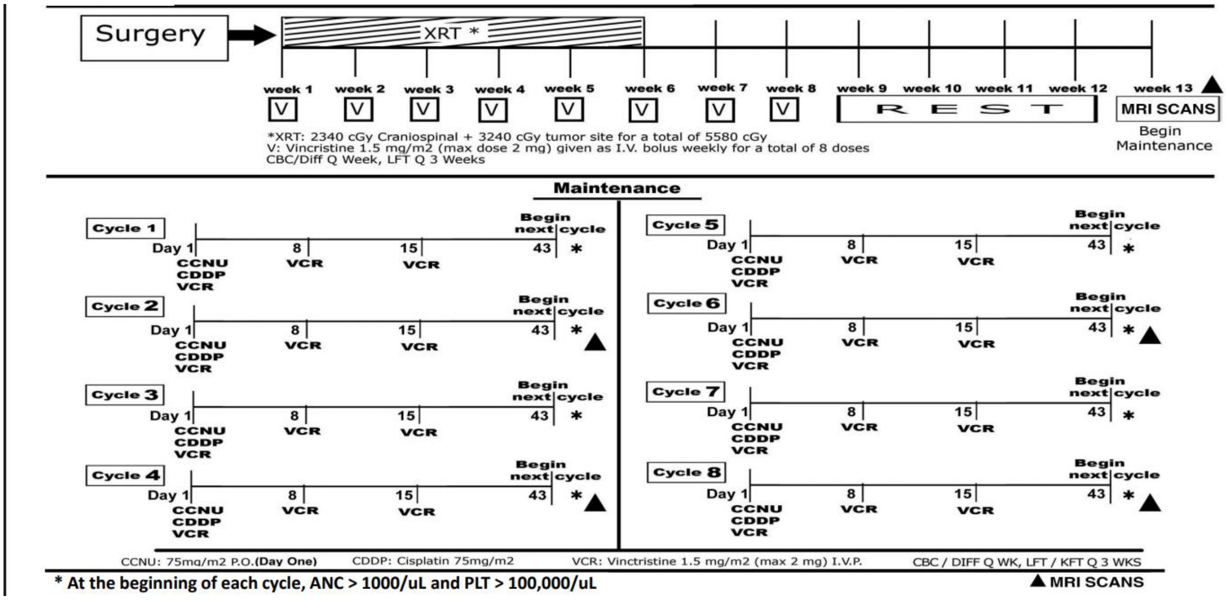

Supplementary Fig. 1 Roadmap for A9961 Protocol, Regimen A

Surgery			Chemoradiotherapy								Maintenance								
	31 Days		Radiation Therapy (XRT)							Rest									
		Cycle									1	2	3	4	5	6	7	8	9
		Week	1	2	3	4	5	6	7		11	17	23	27	33	39	43	49	55
		Day	1	8	15	22	29	36	43										
				Chemotherapy							Maintenance Chemotherapy								
				V	V	V	V	V	V		A	A	B	A	A	B	A	A	B

Maintenance

Cycle A (42 Days)

- CISplatin (75 mg/m^2) IV over 6 hours on Day 1
- Lomustine (CCNU) (75 mg/m^2) orally on Day 1
- VinCRIStine (1.5 mg/m^2, maximum dose 2 mg) IV push or infusion Days 1, 8, and 15

Cycle B (28 Days)

- Cyclophosphamide (1000 mg/m^2) IV over 1 hour on Days 1 and 2
- VinCRIStine (1.5 mg/m^2, maximum dose 2 mg) IV push or infusion on Days 1 and 8
- MESNA (360 mg/m^2/dose) IV infusion over 15-30 minutes starting 15 minutes prior to or at the same time as cyclophosphamide and repeated at 4 and 8 hours.

Supplementary Fig. 2 Chemotherapy of ACNS0331 Protocol [1]

Supplementary Table 1 Comparison of Grade ≥3 Toxicities Between Both Cohorts

Toxicity Type (grade≥3)	All Patients n=168 (%)	Cohort A n=112 (%)	Cohort B n=56 (%)	P value
**Ototoxicity**	29 (17.3%)	27 (24%)	2 (3.6%)	<0.001
**Neurotoxicity**	36 (21.4%)	29 (26%)	7 (12.5%)	0.046
**Nephrotoxicity**	48 (28.6%)	36 (32%)	12 (21.4%)	0.15
**Hepatotoxicity**	31 (19%)	23 (21%)	9 (16%)	0.3
**Leukopenia**	162 (96.4%)	107 (96%)	55 (98%)	0.3
**Thrombocytopenia**	109 (64.9%)	80 (71%)	29 (52%)	0.04
**Anemia**	109 (64.9%)	80 (71%)	29 (52%)	0.04
**Fever & Neutropenia**	79 (47%)	42 (38%)	37 (66%)	<0.001

Supplementary Table 2 Incidence of grade ≥3 ototoxicity every 3 maintenance cycles

Timing of Ototoxicity (Grade ≥3)	All patients	Cohort A	Cohort B	P value
**Ototoxicity, any time point**	29/168 (17.3%)	27/112 (24%)	2/56 (3.6%)	<0.001
**Ototoxicity, cycles 1-3**	5/156 (3%)	4/109 (3.6%)	1/47 (2%)	0.62
**Ototoxicity, cycles 4-6**	21/154 (13.6%)	20/102 (19.6%)	1/52 (1.9%)	0.003
**Ototoxicity, cycles 7-9**	19/142 (13.3%)	17/90 (18.9%)	2/52 (3.8%)	0.01

Reference:

[1] Michalski JM, et al. Children's Oncology Group Phase III Trial of Reduced-Dose and Reduced-Volume Radiotherapy With Chemotherapy for Newly Diagnosed Average-Risk Medulloblastoma. J Clin Oncol. 2021;39(24):2685. https://doi.org/10.1200/JCO.20.02730.
